# Exploring the Experiences of Cancer Patients With Chemotherapy-Induced Ototoxicity: Qualitative Study Using Online Health Care Forums

**DOI:** 10.2196/10883

**Published:** 2019-03-14

**Authors:** Stephanie E Pearson, John Taylor, Derek J Hoare, Poulam Patel, David M Baguley

**Affiliations:** 1 Nottingham Biomedical Research Centre National Institute for Health Research Nottingham United Kingdom; 2 Hearing Sciences Division of Clinical Neuroscience, School of Medicine University of Nottingham Nottingham United Kingdom; 3 Nottingham University Hospitals NHS Trust Nottingham United Kingdom; 4 Academic Unit of Oncology Division of Cancer and Stem Cells School of Medicine, University of Nottingham Nottingham United Kingdom

**Keywords:** quality of life, neoplasms, drug-related side effects and adverse reactions, hearing loss, tinnitus, online social networking, internet, eHealth, social support

## Abstract

**Background:**

Many cancer patients and survivors experience permanent and life-debilitating effects, such as ototoxicity, from treatment. Ototoxicity manifests as high-frequency hearing loss and tinnitus, which can have a detrimental effect on the quality of life (QoL) of those affected. Currently, there is little information and support offered to these patients who experience ototoxicity, potentially leading to many being undiagnosed and untreated.

**Objective:**

The aim of this study was to explore the extent of ototoxic side effects, such as hearing loss and tinnitus, and their impact on cancer patients following chemotherapy treatment. Secondary objectives included detecting the time periods of onset and duration of the ototoxicity and identifying what support was available to this population.

**Methods:**

Posts from publicly available online forums were thematically analyzed using the guidelines by Braun and Clarke. A coding manual was iteratively developed to create a framework for the analysis of the ototoxicity experience among the cancer population.

**Results:**

A total of 9 relevant online forums were identified, consisting of 86 threads and 570 posts from 377 members. Following the *bottom-up* thematic analysis, 6 major themes were identified: *nature of ototoxicity, time of experienced ototoxicity, information on ototoxicity, quality of life, therapies,* and *online social support*.

**Conclusions:**

There was a significant number of reports expressing concerns about the lack of information on the risk of ototoxicity. More support for those suffering is needed; for example, improved interdepartmental communication between oncology and audiology services could optimize patient care. Patients should also be encouraged to communicate with their health care professionals about their ototoxicity and relay how their QoL is impacted by ototoxicity when accessing support. Tinnitus was the most common concern and was associated with distress. Hearing loss was less common; however, it was associated with fear and employment issues. Those who reported preexisting conditions were fearful about worsening their condition as their QoL was already impacted.

## Introduction

### Background Information

Although there are an estimated 17.5 million cancer cases per year worldwide, the development of screening programs and improved diagnostics have contributed to an increase in survival rates [1,2]. The current overall 5-year survival rate is 67%, this means that many more cancer survivors are now living with the late effects of cancer treatment, such as peripheral neuropathy and ototoxicity [3]. Ototoxicity is defined by American Speech-Language-Hearing Association as a decrease in hearing thresholds relative to baseline testing and indicates evidence of damage in hearing caused by medication [4]. Platinum-based chemotherapy, for example, cisplatin, although a highly effective antineoplastic agent, is known to cause peripheral neuropathy and ototoxicity (resulting in tinnitus and hearing loss) [5]. Tinnitus is defined as the manifestation of a conscious perception of an auditory sensation without a corresponding external stimulus [6,7].

These effects can potentially have a significant impact on quality of life (QoL). Tinnitus, for example, is associated with sleep difficulties, and hearing loss is associated with dementia [8,9]. A deeper understanding of the impact these long-term consequences of cancer treatments can have on QoL can improve long-term symptom management in patients living with the debilitating effects of cancer treatment [10,11].

There is a lack of information on the prevalence and effect of ototoxicity because of the underreporting of ototoxic events, and few longitudinal studies have been carried out [12,13]. The literature advising on the diagnosis, grading systems, and management of ototoxicity is heterogeneous across studies, resulting in poor-quality information being available [13-15]. Consequently, this has had a substantial effect on the quality of support offered to patients, as there is no standard protocol or guidance for clinicians to follow.

The increase in popularity of using the internet allows anyone to access health care information instantly and can potentially improve patients’ knowledge and help with treatment decisions [16,17]. Technological advances have meant that this method of research is becoming increasingly used within medicine [18]. Although this methodology has not been used for ototoxicity, there have been thematic analyses carried out on Web-based group discussions in Parkinson disease and men’s fertility issues [19,20]. There has also been, although inconclusive, evidence to show that Web-based support for cancer patients has a positive effect [21]. By exploring online health care forums (OHFs), the impact on QoL from ototoxicity can be analyzed. OHFs are a way in which patients can contribute to a range of personal health-related discussions openly with one another by grouping various threads on a specific topic [16,22,23]. Individuals suffering from long-term effects of treatment are significantly more likely to participate in this Web-based community to discuss health concerns [24].

### Aims and Rationale

Approximately 14.2% of long-term cancer survivors live with disabilities directly caused by their cancer treatment and its toxic nature [10,25]. Clinical reports of patients may not reflect the true incidence or severity of the late effects caused by treatments, specifically ototoxicity. In many cases, patients ask medical questions or share experiences on the Web that they could not in person. Yet this potentially rich source of information has not yet been explored. Thus, the aim of this study was to explore the true demographic and the impact on QoL of ototoxic effects caused by cancer treatment via analysis of OHF discussions. The secondary objectives were to explore the time course of ototoxicity occurrence in relation to treatment, whether the adverse effects were reported as temporary or permanent, and which means of support patients had access to and used. This research has the potential to significantly inform clinical and social aftercare of those who have been treated for cancer.

## Methods

### Ethical Considerations

Ethical approval for the study was obtained by the University of Nottingham, School of Medicine Ethics Committee. Although informed consent was not required in this study as the information was available in the public domain, all members’ personal details were kept anonymous to maintain confidentiality and protect privacy [26].

Quotes used were extracted as part of a longer original post. Details that would allow the member to be traced were excluded. Risk to forum users was deemed to be minimal.

### Sample and Inclusion Criteria

Relevant and representative forums were identified using the 4 most common search engines: Google, Yahoo!, AOL, and Bing [27]. Search terms included combinations of “impact,” “effect,” “forum,” “discussion,” “hearing loss,” “tinnitus,” “chemotherapy,” and “cancer.” Inclusion criteria were (1) forum did not require membership (ie, publicly available) and (2) the forum content was in the English language [28]. The first search page was screened for results, and there were no date restrictions for the searches. Relevant OHFs were manually extracted onto Excel, and the threads within these OHFs were screened. A thread was considered relevant when the post itself mentioned hearing loss or tinnitus and either cancer or chemotherapy, by asking a question or offering support. The relevant threads were extracted ready for thematic analysis.

### Thematic Analysis

**Figure 1:**
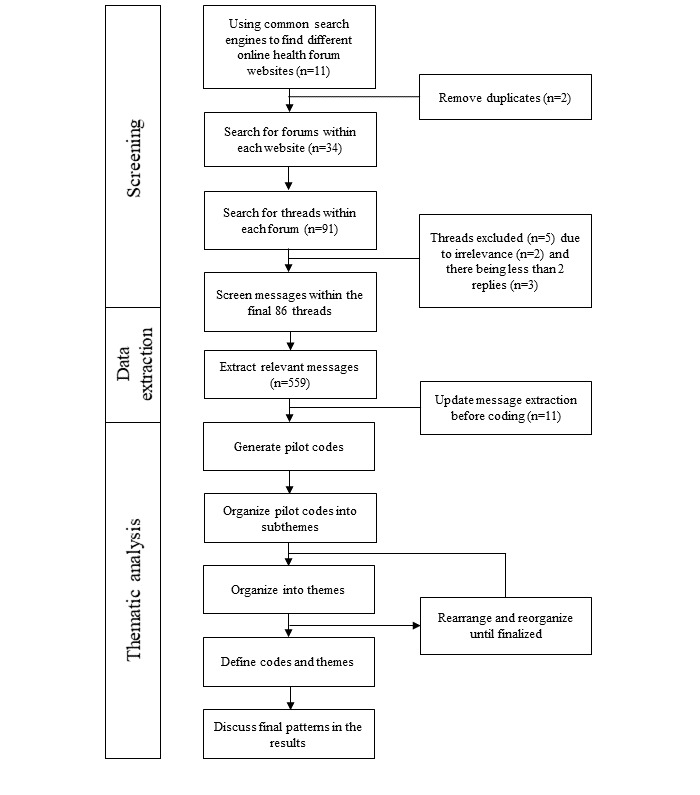
Flowchart representing the methodology and strategy of the online health care forum screening and analysis.

The data extraction and the thematic coding strategy were based on the Gao et al [16] and Braun and Clarke [26] methodologies. Whole threads were screened, and the messages deemed irrelevant or which had too few replies were excluded (Figure 1). Messages were then extracted for thematic analysis. The number of members posting on the forums was quantified and the threads were randomized using computer software.

Following the *bottom-up* strategy, open coding was performed by making initial comments on the first 100 messages, from which a pilot coding manual was produced. For example, the quote “The most worrying thing I have come across is that I may suffer changes to my hearing, I am petrified.” was described as a fear of developing ototoxicity. This was followed by grouping similar descriptions together into codes which fit into categories, and finally, arranging these into clearly defined themes [29]. Using the example quote, this was defined as a *general fear* code and grouped into the *emotions* category in the *quality of life* theme. A total of 2 researchers (SP and JT) independently coded the remaining randomized messages against the coding manual and compared results. Every difference was discussed, and the coding manual was reorganized and developed accordingly. Following this discussion, the 2 researchers (SP and JT) agreed on a final coding manual, and the remaining messages were coded and quantified to assess which topics people discussed most frequently.

## Results

### Description of Included Forums

The search found 11 OHF websites varying in popularity. This was further narrowed to 9 following the elimination of duplicates (Table 1). A total of 34 OHFs were identified, and 86 threads were included in the final analysis. The number of messages within the threads varied greatly. For the larger threads, only the most relevant messages were extracted. Over 3000 messages were screened, and a total of 570 messages were manually extracted for the final analysis.

The numbers of members in each thread posting about ototoxicity ranged from 1 to 17, with 56 members seeking information and sharing their experiences in multiple threads and forums. The forums themselves varied in popularity; however, the number of active members was not always publicly available. Overall, 377 members were responsible for the 570 messages extracted (Table 1). The geographical information of the posts was not always available though it consisted mainly of the United Kingdom and United States; however, there were also threads based in Australia, New Zealand, and South Africa.

### Thematic Analysis

**Figure 2:**
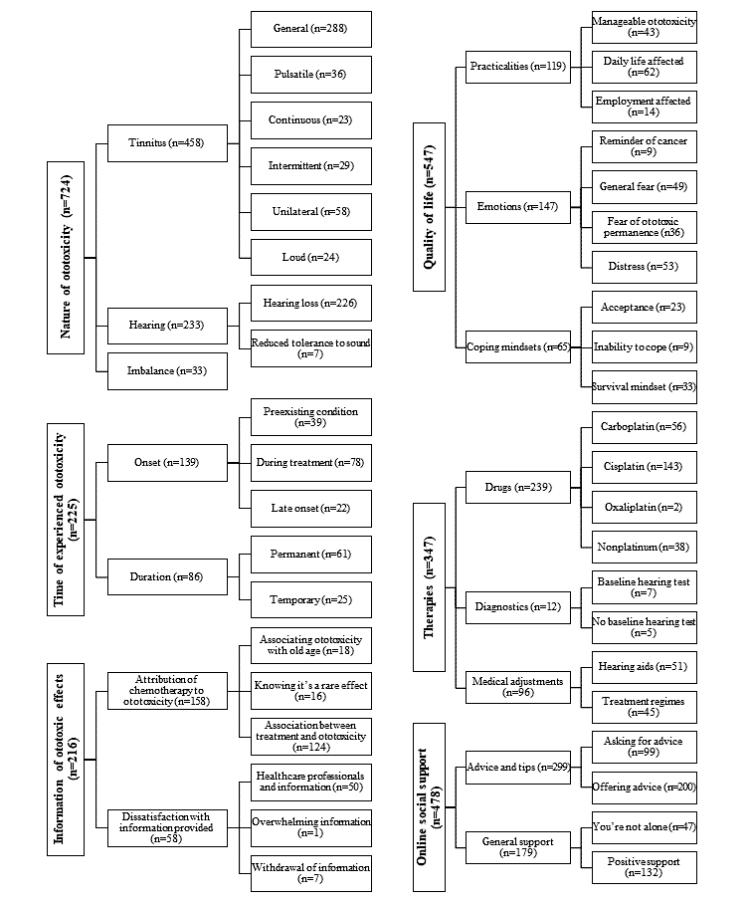
The themes, subthemes, and codes found within the forum messages and the number of times the codes were reported (n). Themes include the nature of ototoxicity (n=724), time of experienced ototoxicity (n=225), information on ototoxicity (n=216), quality of life (n=547), therapies (n=347), and online social support (n=478).

A total of 42 final codes were generated from which to interpret the forum messages by following the Braun and Clarke methodology (Figure 2). The names of each category and theme emerged through discussion of words and terms that the 2 researchers thought reflected the set of codes, which were then reviewed by all authors. The 6 overarching themes were as follows: (1) Nature of ototoxicity, (2) Time of experienced ototoxicity, (3) Information on ototoxicity, (4) Quality of life, (5) Therapies, and (6) Online social support.

**Table 1:** The number of threads extracted, the number of messages extracted, and the range and total of participants and members posting within the online health care forums.

Forum names	Threads analyzed (n)	Messages analyzed (n)	Participants in each thread (n)	Members posting in multiple threads (n)	Members in OHFs^a^ (n)
Forum 1	8	105	1-16	6	50
Forum 2	5	41	3-10	4	32
Forum 3	18	122	2-12	14	69
Forum 4	9	68	3-17	11	49
Forum 5	19	80	1-5	7	55
Forum 6	5	14	1-8	1	14
Forum 7	8	37	2-7	6	27
Forum 8	3	21	4-12	1	20
Forum 9	11	82	3-14	6	61
Total	86	570	1-17	56	377

^a^OHF: online health care forum.

## Discussion

### Nature of Ototoxicity

The *nature of ototoxicity* theme was the most discussed within the forums (n=724). It consisted of describing and categorizing the ototoxic symptoms experienced. This theme consisted of the following subthemes: *tinnitus (n=458), hearing* (n=233), and *imbalance* (n=33). The *tinnitus* subtheme entailed the personal perception of tinnitus, as described by the members. Approximately 80.4% (458/570) of the messages within the forums reported some experience of tinnitus, suggesting that tinnitus is a significant occurrence within this population:

I reported to my oncologist that I experience the loudest high pitched ringing that makes me put my hands over my ears!

User X, Forum 7

The most common aspects of tinnitus described, in addition to a ringing sensation (*general tinnitus n=288)*, were *pulsatile tinnitus (n=* 36) *continuous tinnitus (n=23)*, *intermittent tinnitus (n=29)*, *unilateral tinnitus (n=58),* and *loud tinnitus (n=24).* Users reported *pulsatile tinnitus (n=36)* as a *heartbeat thumping* or *whooshing sounds* in the ear. *Pulsatile tinnitus* —although no known research has been conducted on the association with cancer treatments—has been thought to be of a vascular origin and can be synchronized with the heartbeat [7,30]. *Unilateral tinnitus* seemed to occur more in the left ear (n=34) compared with the right ear (n=24). Previous research reported approximately 15% of patients treated with cisplatin had experienced unilateral tinnitus [31]. A similar total of 13% of forum messages mentioning *tinnitus* were found to be *unilateral tinnitus*, which correlates with the literature:

I just came out of the ward after my first treatment cycle, I feel okay in myself apart from this constant high ringing in my left ear.

User A, Forum 4

Within the *hearing* subtheme, *hearing loss* (n=226) was commonly reported. Only 7 members reported *reduced tolerance to sound*. *Hearing loss* was mentioned by users before receiving treatment and being fearful of the deficit, in addition to complaints of already experiencing hearing loss because of chemotherapy. Furthermore, multiple users expressing hearing loss mentioned already having a form of hearing deficit that had worsened. Although there has been research carried out reporting hearing loss following chemotherapy, it is worth noting that *reduced tolerance to sound* has not previously been reported in the literature as an adverse effect, and as such, further research is warranted [32-34]:

The other day I had a hearing test which confirmed that cisplatin has damaged my hearing. I can no longer hear well

User Y, Forum 7

Within the OHFs, there were also members reporting *imbalance,* which were mainly discussed in threads associated with breast cancer (n=33). *Imbalance* seemed heavily associated with Femara and Taxol treatments. Vertigo has been reported in a study investigating the etiology in 36 breast cancer survivors; however, it is rarely associated with ototoxicity within medical literature [35]:

I am currently getting weekly Taxol treatments with Carboplatin. I have had acute episodes of sudden vertigo. Both times I was reading a book and suddenly felt the room spinning. The feeling lasts about 10 seconds but is very intense! I have no previous history with vertigo.

User C, Forum 1

### Time of Experienced Ototoxicity

The *time of experienced ototoxicity (n=225)* was variable within the OHFs. Although members described their *onset* as *preexisting before treatment* (n=39), *during treatment* (n=78), or *late onset* (n=22), specific times were also noted. For example, there were reports of tinnitus and hearing loss occurring from the first cycles (n=9, n=5, n=3 for cycles 1, 2, and 3, respectively) to occurring 2 years following treatment. It is thought that pre-existing hearing deficits increase the risk of experiencing ototoxicity [36]. However, only 28.1% (39/139) of those mentioning the onset of ototoxicity admitted to having prior hearing deficits [37]. Those with preexisting conditions mainly shared their concerns about further damage:

This is worrying me, I am due to start treatment but I already have congenital severe hearing loss which is corrected by the use of hearing aids. The thought that chemo could make things worse is a real issue for me. The thought of chemo is scary enough but the thought of further damage to my hearing is scarier still.

User B, Forum 2

Many members believed tinnitus would be temporary and expressed shock when it became permanent. Furthermore, members sought validation that what they were experiencing was an adverse effect and not the reoccurrence of cancer. From those who reported their *duration* (n=86) of ototoxicity, 71% (61/86) reported having *permanent* tinnitus:

It’s just over 10 months since I finished treatment. I had bad tinnitus during treatment but it completely disappeared for months. I've noticed that in the past few weeks my tinnitus is back intermittently maybe 2/3 times a day. Has anyone else had tinnitus reappearing after months and months of nothing?

User Z, Forum 5

It is worth noting that many users did not share the duration of their tinnitus. Further research is needed to identify possible risk factors of permanent tinnitus, even though it is known that tinnitus caused by chemotherapy is associated with age and a higher accumulative dose [5,38-41]. Users did not commonly report their age or dose of treatment; therefore, it would be difficult to conclude if this population reflects previous studies. Overall, members appeared to remain positive, frequently sharing tips and advice to help others, despite posting how permanence of tinnitus affected their QoL:

My overall hearing loss has been profound but it’s a side effect of Cisplatin I'm afraid. I'm not expecting it to go away, and I've been told it's likely to be permanent. Sorry not to be more positive.

User C, Forum 5

### Information on Ototoxicity

One of the discussion topics reported throughout the different forums was the *information on ototoxicity* (n=216). This theme consisted of the *attribution of chemotherapy to ototoxicity* (n=158) and *dissatisfaction with the information provided* (n=58). The *attribution of chemotherapy to ototoxicity* consisted of members who associated hearing loss with *old age* (n=18) or believed that because it is a fairly *rare* (n=16) toxic effect of chemotherapy, there must be another cause. This could be because of denial from the patients or simply a lack of knowledge on ototoxicity:

These days I have ringing in my ears. It’s probably due to my old age.

User A, Forum 9

The majority of members, however, did see an *association between chemotherapy and ototoxicity* (n=124). The members who had more knowledge on ototoxicity were vocal in sharing what they had been told by health care professionals, urging those who had not made the association to see an audiologist or their general physician. Furthermore, many members expressed some *dissatisfaction with the information provided* (n=58). Members shared their anger, disappointment, and *dissatisfaction with health care professionals* and *lack of information* (n=50) because they had not been warned about ototoxicity:

Straight after my first cycle, I started suffering from a ringing noise in both ears. I told my oncologist about it and she just made a note, and sent me off for the third dose without saying a word! I was not warned about this and don't get the impression that anyone cares other than me.

User C, Forum 2

There appeared to be a lack of communication between patients and professionals, which was reported frequently throughout the forums, despite there being a significant number of studies aiming to raise awareness of ototoxicity [42]. Members expressed having felt ignored and not taken seriously during consultations:

I think they don't reveal all of these things to us because they don't want to scare us away from doing the chemo. When I first started having tinnitus all I got was “I've never seen a case from carboplatin” like I'm making it up. I looked back through all my papers they gave me at first for the side effects and there was one notation about a rare side effect: 'hearing changes'. I really feel like I didn't have all the information I needed at the time I was making my decisions. It does make you wonder what else they haven’t told us.

User W, Forum 1

In contrast, members also confessed to *withdrawal of information* (n=7) by lying or not telling their clinicians about the severity of their tinnitus and hearing loss because they feared having to compromise the dosage of their chemotherapy and feared morbidity. This could have detrimental impacts on both patients and clinicians as it reduces any reliable information reported:

I’m worried that if I tell the truth about the months of diarrhoea and headaches and tinnitus and the newer extreme tiredness, they'll say I'm too old and fragile to get any more treatment and dump me from the trial.

User Y, Forum 1

There was only 1 message expressing how *overwhelming information* (n=1) stopped them from listening to the information provided, leading to that individual becoming fearful of undergoing treatment.

### Quality of Life

Another main theme was the severity of ototoxicity and the impact this had on *quality of life* (n=547). Members discussed *practicalities* (n=119), *coping strategies* (n=65), and *emotions* (n=147) associated with how their *quality of life* was compromised. Numerous messages implied the symptoms were mild, with many members saying they had *manageable symptoms* (n=43) that they could easily cope with or ignore, such as:

Most of the time when I am busy, I don't notice it [tinnitus], but as you probably know, when you become aware of it, it is hard to ignore. I hope you are lucky and yours goes away.

User H, Forum 5

However, most users reported in abundance how their *day-to-day life* (n=62) was affected by ototoxic effects. Many members shared concerns over how their hearing loss affected their relationship with their partners and family members, which could be distressing and isolating:

I'm three years post chemo and now have tinnitus in my left ear which is getting worse. I don't recall being told chemo could damage ear and it drives me mad. Will it ever go? Sound sets it off so if I sit in silence its okay but it's affecting my relationship now. I don’t know what to do.

User F, Forum 2

Research has been conducted on how tinnitus and hearing loss affects QoL; however, it has not been expanded into the population facing cancer treatment and survivorship [43-45]. In addition, the few studies exploring QoL affected by tinnitus do not go in depth into what aspects of life ototoxicity affects [6,46]. Therefore, by exploring these messages from the forums, specific aspects of QoL affected by ototoxicity can be identified. This will help develop a relevant and tailored support system for these patients:

I cannot hear at all in my left ear ever since having chemotherapy. I have been fitted, aged 39, with a hearing aid but I have very short hair as a result of chemo, so they show and it affects my self-esteem. I don't sleep with the aids in so I can’t hear my baby when she wakes at night which I find really distressing. I feel about 90.

User S, Forum 2

Another concern among members was the effect ototoxicity had on *employment* (n=14). Specifically, professional musicians shared their fear over losing the ability to play music. Members spoke of the risk of losing their hearing being catastrophic for their employment and even mentioned early retirement. Most of the questionnaires used to assess QoL and ototoxicity do not mention the impact on employment. This area needs to be explored clinically, specifically in those who critically rely on hearing to be employed, such as musicians:

Better than dead? At this point, I'm wondering. I cannot work with this condition because my job requires proper hearing. Hearing loss and this constant tinnitus is life-changing, far more than having cancer is. This has me worried more than living with cancer. I'm wondering if I'll ever have another day where I can hear clearly and be a productive member of society.

User Z, Forum 4

Of the main issues faced with ototoxicity, one was how it acted as a *reminder of cancer* (n=9). Although members mentioned successfully managing the tinnitus and hearing loss, it acted as a permanent reminder of what difficulties they had been through. There was a sense of *general fear* (n=49) experienced across the forums, as members frequently discussed being fearful of losing their hearing and how this could affect their life. In fact, many people discussed concerns over safety and how this gave them anxiety. As mentioned previously, these aspects of life are rarely included in questionnaires, and therefore, are rarely reported in the literature:

I cannot hear the door opening, food cooking, the television or radio and comprehend what they're saying. It's dangerous. I never realized how much we rely on the sounds of cooking. No more multi-tasking in the kitchen, I have to stand and watch the stove top now.

User A, Forum 9

Within this sense of fear, there was a specific *fear of permanence* (n=36) of the ototoxic effects. There were frequent concerns over how long the hearing loss and tinnitus would last and if they would ever recover normal hearing. Currently, there is little knowledge about the duration of ototoxicity and no predicting factors, which could further induce this fear in patients:

I completed all of my chemotherapy cycles and since then I have lost a lot of hearing and also have ringing in my ears. Has anyone experienced this and gained hearing back? I am hoping since I only finished a month ago I will improve, but no one is telling me anything. If anyone has a positive story I would love to hear about it, to give me hope.

User F, Forum 3

Associated with fear was *distress and severe impact on QoL* (n=53). There were messages that described hearing loss and tinnitus as “unbearable, severe and extremely bothersome,” which is consistent with the current literature on how tinnitus and hearing loss affect QoL in the general population [47]. However, managing chemotherapy-induced ototoxicity in addition to coping with cancer can be extremely distressing; therefore, appropriate multidisciplinary support should be considered urgent:

Tinnitus is controlling my life right now and I don’t know what to do. I am suicidal and keep thinking of the best way to end this misery once and for all. I don’t know how long I can keep this up. I wish I was strong like all of you in this forum but I am so weak and fragile right now. I gave up on God ever existing cos if he did exist then none of us would be suffering like this right now and diseases such as cancer would not exist.

User E, Forum 6

How members coped with ototoxic effects varied greatly throughout the forums. A total of 3 codes formed the *coping mindsets* subtheme: *acceptance of ototoxicity* (n=23), *survival mindset* (n=33), and the *inability to cope* (n=9). Messages on *acceptance* and having to *learn to live with tinnitus* varied from being positive to resentful:

I'm afraid I do not know how to say this without being blunt, but would you really rather die than live with some permanent tinnitus from your cancer? Most of us have a few souvenirs from cancer, I think that is better than dying.

User G, Forum 4

I finished all my chemotherapy 6 months ago and I am still suffering from side effects. Numbness in my fingers and toes, pain in my feet and calves and hearing loss. I AM happy to be alive, but I can't shake off the dissatisfaction I have with the body treatment left me with.

User D, Forum 3

The most frequent *coping mindset* was the *survival mindset*. People shared thoughts such as “worry about the cancer now and the side effects later” within this *survival mindset* and tended to promote this view of ignoring any side effects until after the cancer was in remission. This mindset could in fact be partially responsible for the underreporting of toxicities in clinical trials, therefore, having negative clinical implications on research. Patients should be encouraged to speak openly about their experienced toxicities:

The most important thing is that the chemotherapy worked, it just seems silly for us to be moaning about a bit of tinnitus.

User P, Forum 5

Although not many, there were members with an *inability to cope*. These members appeared to be extremely depressed and seemed to need urgent care and advice, such as counseling:

I have had the Cisplatin dose reduced 20 percent for the second round due to the ringing and hearing loss. I can't seem to find anything positive to report. Most say it’s permanent, including my oncologist and audiologist. I may be forced to stop treatment if mine gets any worse because I'd rather be dead than deaf.

User I, Forum 4

By supporting these patients before their tinnitus and hearing loss worsens, the health service and patients alike could potentially save on mental health costs. Moreover, aspects such as sleep, employment, and relationships are all major parts of life, and when these are affected, it can have a devastating effect on a whole population. It has been reported that those with more comorbidities seem to experience a higher incidence and severity of tinnitus, which could factor in to having a lower QoL [46]. Research is needed to predict and identify patients who need more support to prevent this detrimental effect on their mental health.

### Therapies

Members discussed which *drug* (n=239) treatment regimens they were on, such as *cisplatin* (n=143)*, carboplatin* (n=56)*, oxaliplatin* (n=2), and *nonplatinum drugs* (n=38). Some messages expressed simply their regime and their adverse effects, without mentioning how it affected their QoL. It is difficult to conclude how these members cope and are affected by ototoxicity:

I have severe hearing loss and osteoporosis from carboplatin. I am only 21.

User G, Forum 3

There were few members discussing the *diagnostics* (n=12) they experienced, with an almost equal number of members stating they had *no baseline test* (n=5) compared with having had a *baseline test* (n=7). This correlates with studies having found that baseline tests are not as frequently carried out as suggested [48]:

I had a baseline reading before chemo. Showed mild age related hearing loss, but I could still hear compared to now!

User H, Forum 9

I didn't have a baseline test before starting chemo because no one suggested it.

User L, Forum 4

A subtheme that was relatively abundant within the OHFs was *medical adjustments* (n=96). This involved many of the members having to wear *hearing aids* (n=51) because of the ototoxic effects, and others *adjusting treatment regimens because of ototoxicity* (n=45), which involved anything from changing the drug and lowering the dose to stopping treatment altogether to prevent any further hearing loss or tinnitus. This code was heavily associated with *fear of permanence* and *distress*:

The hearing test I had told the audiologist it was permanent. I got hearing aids about 3 months after treatment was over. I wasn't told hearing loss was a possibility. Every time I went to a doctor, I asked the doc to look at my ears. Finally, one said, see the audiologist. I was crushed - I was only 45 at the time. I did get the hearing aids, and they help so much.

User C, Forum 3

### Online Social Support

Finally, there was a sense of *online social support* (n=478), which included support expressed by members to create a community and develop friendships. From the 570 different forum messages, only 1 message was interpreted as negative. The *advice and Tips* (n=299) subtheme involved members *asking for advice* (n=99) and *offering general advice* (n=200) from how to ignore tinnitus to which hearing aid to use. There were many messages that offered *positive support* (n=132) and used terms such as “you’re not alone” (n=47):

I hadn't realised how many others have developed Tinnitus too—nice to be in good company.

User D, Forum 9

A significant number of patients expressed concerns over not being adequately informed about the true risk of ototoxicity. Although some members expressed having been warned of the risks, they reported that the information given to them was vague and unclear. Some spoke about referrals to audiology departments and seemed satisfied with this level of support. It could be suggested that more interdepartmental communication be made to optimize patient care. Furthermore, information available to patients on ototoxicity could be improved by updating the chemotherapy leaflets. Patients may feel overwhelmed with the amount of information given to them; thus, the information should be shared on a case-by-case basis. It is noteworthy, however, that from the 570 messages analyzed, only 1 message expressed feeling overwhelmed by the information about ototoxicity.

Members who were fearful of losing hearing were those who had preexisting conditions. This could be because their QoL has already been impacted, whereas those posting who had no previous experience with hearing loss or tinnitus would not know how exactly their QoL may be affected. For many, ototoxicity is not an immediate concern when thinking of chemotherapy. However, once the immediate adverse effects subside, ototoxicity remains as a distressing reminder of their cancer. Patients should be encouraged to communicate with their health care professionals about their ototoxicity and relay how their QoL is impacted to access the appropriate support.

There were more reported concerns over tinnitus than any other ototoxic effect. Tinnitus was also associated with distress and the inability to cope. Members posted concerns over sleep, their relationships, and their mental health. More clinical interventions, such as cognitive behavioral therapy, should be readily available to this population. Furthermore, hearing loss was common within the OHFs but was more associated with fear of losing hearing, fear over personal safety, and fear of hearing loss impacting employment.

### Limitations of This Study

As this research was observational and exploratory, there was no way of quantitatively measuring QoL. The posts were subject to misinterpretation, even though 2 researchers were involved in analysis to minimize the risk of this potentially occurring. Although OHFs are popular within communities, this sample is not necessarily representative of the population, as only 2 of the forums had information on number of members and active threads; therefore, it is difficult to draw conclusions on the exact population. Only those who have internet access and are inclined to voluntarily share personal information participated. It is also possible that those who post on forums are those with the most severe worries, and those whose questions have not been answered by health care professionals, meaning they seek advice on the Web. Furthermore, gender, age, and geographical location of the members were mainly unknown. Therefore, no analysis could be undertaken on these factors.

### Conclusions

In conclusion, ototoxicity has a significant burden on the QoL of those suffering from cancer. More information and support should be available to this population to help manage these long-term symptoms. Tinnitus was the most frequently reported ototoxic effect within the OHF, followed by hearing loss. The ototoxic effects were associated with lower QoL, fear, isolation, depression, and frustration that patients were not warned enough about these effects.

## Abbreviations

OHF: online health care forum
QoL: quality of life
